# The Immune System and the Plural Autisms: A Narrative Review

**DOI:** 10.3390/jpm16070379

**Published:** 2026-07-15

**Authors:** Paul Whiteley, Kevin Carr, Paul Shattock, Malcolm Hooper, Carol Stott, Karl Hardy, Ben Marlow, Chandoshi Rhea Mukherjee, Athena Whiteley

**Affiliations:** 1 ESPA (Education & Services for People with Autism) Research, North House, Ferryboat Lane, Castletown, Sunderland SR5 3RL, UK; 2 ESPA (Education and Services for People with Autism), North House, Ferryboat Lane, Sunderland SR5 3RL, UK; 3 BeginningwithA, 36 Mill Road, Cambridge CB25 9EN, UK; 4 Synapse Centre for Neurodevelopment, Colchester Hospital, East Suffolk and North Essex NHS Foundation Trust, Colchester CO4 5JL, UK; benjamin.marlow@esneft.nhs.uk; 5 Worcestershire Acute Hospitals NHS Trust, Worcester WR5 1DD, UK; 6 Independent Researcher, Sunderland SR5 3RL, UK

**Keywords:** autism spectrum disorder (ASD), immune system, comorbidity, screening, intervention

## Abstract

Plural autisms offer one important way of organising the massive heterogeneity that is currently included under the singular diagnostic label of autism spectrum disorder (ASD). Characterised exclusively by behavioural criteria related to social communication skills and restricted or repetitive patterns of symptoms, the observable diversity in presentations and developmental trajectories partially explains the lack of universally applicable biomarkers and answers about underlying biology. One particularly important area potentially pertinent to several manifestations of the plural autisms is a connection to the immune system, whether noted across differing patterns of immune functions or following immune challenge in the context of inflammatory processes exerting an effect on developmental processes and behaviour. Utilising a narrative review, we highlight various research analysing a role for immune functions in the context of the heterogeneous autisms stretching across under-, over- and autoimmune processes. Current evidence for immune system involvement in various autisms carries considerable limitations, and studies are often based on small sample sizes, focusing on selective clinical subgroups, and using case-based observations. Notwithstanding the available evidence, substantial and more detailed further studies are required in relation to immune-related screening, building also on the currently limited evidence on the potential usefulness of personalised immune-affecting interventions following appropriate screening and identification of pertinent immune-related issues.

## 1. Introduction

Defined exclusively by the presence of issues with social communication and social understanding alongside the appearance of restricted and/or repetitive behaviours meeting defined diagnostic cut-off points [1,2], autism or autism spectrum disorder (ASD) is a label that continues to confound. Formally defined in 1943 [3], massive efforts have subsequently gone into determining the potential cause(s) of autism, with various aims to, for example, predict autism, engineer an objective test or tests for autism, and provide definitive data on interventions that positively affect life outcomes for those diagnosed. With huge resources being invested, science is making some progress, albeit with obstacles remaining. We are currently for example, still reliant on overt behaviour for assessment and diagnosis (alongside detailed professional analysis of developmental history); we cannot reliably predict who will become autistic and, indeed, who will remain autistic across a lifetime; life outcomes for those diagnosed with autism as a singular diagnostic grouping have not significantly shifted in quality in the decades since formal recognition by Kanner [3]. Although there are various reasons to explain why such issues have not been suitably tackled, one particularly important variable stands out: despite being united by the presence of the diagnostic criteria for autism, remarkable heterogeneity is an important and enduring facet of autism [4].

Such heterogeneity in presentation is seldom seen in other medical, developmental, and psychiatric conditions. Whilst guided by core criteria for autism, the variability in the presentation of those criteria is vast. It stretches from those with high-support-needs autism, who are reliant on daily help and support to accomplish basic tasks, to those who are seemingly able to traverse the social world with minimal need for help and support to meet their daily needs. This heterogeneity is made even more complicated by the presence of multiple and similarly heterogeneous somatic and psychiatric comorbidities occurring alongside autism [5]. Combined with potential non-static presentation(s) of autism, governed by biological variables such as maturity alongside other biopsychosocial influences, the recognition of just how complicated autism as a singular label is comes into full view.

We have previously offered a potential solution to help tackle the key issue of heterogeneity via the adoption of the concept of plural autisms [6]. We provided evidence for how there are multiple pathways towards a diagnosis of autism and, within this and other definable parameters, how there are important ways that autism can be pluralised to reveal subgroups or, at least, more homogenous phenotypes. Such pluralisation is, we argued, already a facet of the current diagnostic manuals for autism. Both DSM-5 and ICD-11 [1,2] provide scope for diagnosticians to grade the severity of autistic features based on parameters such as verbal language ability, intersecting with newer definitions such as profound autism [7] adopted by the US Autism and Developmental Disabilities Monitoring (ADDM) Network. Combined with listing multiple boundary diagnoses that manifest autistic traits, the ICD-11 autism criteria, in particular, provide plenty of scope for the pluralisation of autism.

Of the multiple potential areas that are seemingly connected to a diagnosis of autism or ASD and/or potentially impact the presentation of symptoms, one particularly important issue stands out: a role for the immune system or immune function responses to a stressor [8]. Herein, we present findings from a narrative review of available studies related to the immune system and autism, highlighting pertinent findings and drawing attention to the often preliminary and heterogeneous character of much of the currently available evidence.

## 2. The Immune System and (Some) Autisms: The Research Beginning

The immune system is traditionally seen to represent the internal suit of armour that defends an organism from the surrounding environment. It is a complicated network of different chemicals, cells, and organs that affords some protection from the multitude of pathogens—viral, bacterial, and fungal—that invade living organisms on an almost constant basis. As an integral part of animal evolution, the immune system has ensured species survival, manifesting as the innate immune system (born with) and the adaptive immune system (developed in response to pathogen exposure).

As knowledge of the immune system—its workings and functions—has increased, the identifying and protecting roles of the immune system have also been expanded. One particularly surprising bidirectional relationship has emerged within the realm of immune functions or response to immune functions, being linked to brain development, developmental psychology, complicated overt behaviours, and vice versa [9]. Stress, for example, brought about by combinations of psychological and/or social factors, can affect immune functions. Precise mechanisms are still being investigated, but various psychosocial stressor events are known to produce a cascade of biological processes that ultimately end either by ‘activating’ the immune system or causing conditions, typically inflammatory conditions, that evoke some kind of immune response. Similarly, various immune functions are known to impact brain and neurotransmitter functions, considering that the central nervous system (CNS) has its own complicated set of immune functions in the form of glial cells, such as astrocytes and microglia. Cumulatively, what such advancements point to is a complicated set of processes by which immune functions can and do affect neurology and behaviour and how certain behaviour(s) can conversely seemingly affect immune functions.

Considering this growing biopsychosocial role for immune functions, many people would not automatically link the immune system with a complicated, behaviourally defined developmental diagnosis like autism. The idea that a system, which at heart is designed to identify and eradicate various types of pathogens, might show some connection to developmentally complex and often long-persisting behaviours is a difficult step to take. We should not, however, be so surprised by this connection. One need only look at the scientific literature on the potential behavioural effects following encephalitis and related post-infection neuroinflammatory conditions to see the immune-behaviour connection at work. Indeed, encephalitis is now listed as a boundary condition in the ICD-11 autism diagnostic criteria with the added context, “*Autistic features may become manifest in the context of acquired medical conditions, such as encephalitis.*” [1], acknowledging the rarity with which such issues probably occur.

The history of examining immune functions in relation to autism is long, complicated, and continually beset by issues of heterogeneity in autism presentations, affecting the relevance and applicability of the scientific evidence presented. Some of the earliest papers on this topic documented a connection between specific infections and some cases of autism, initially focusing on congenital rubella and autism [10]. Autism as an outcome of viral exposure to rubella in utero followed a pattern of different somatic and behavioural outcomes stemming from such disease. As technology advanced and allowed, for example, the measurement of viral titer levels, it also revealed that some children with autism seemed to be potentially at unique risk of lacking the protection afforded by rubella vaccination [11], which potentially may have been part of the effect of the viral pathogen on the onset of autistic symptoms. Such work is also important given estimations on the potential impact of rubella vaccination on averting cases of autism, assuming appropriate acquired immunity [12].

Further studies on the immune system and autism continued, noting in some cases a “*range of immunological injury hypotheses for the genesis of the pervasive developmental disorders*” [13] and further hinting at a connection between immune functions and some cases of autism. Much of the progress coincided with technological advances in relation to both probing immune functions and characterising specific immune cellular tasks and responses. Various types of immune-related issues emerged in relation to cases of autism, focusing initially on areas such as CD4+ helper T cell depression [14], moving onto a role for the complement system and major histocompatibility complex (MHC) [15], and then further to the presence of autoantibodies against brain tissue [16]. Such early research revealed that immune issues coincident with some manifestations of autism covered a multitude of areas across under- and overactive immune system activity and autoantibody-associated immunity, with findings spanning both the innate and adaptive immune system.

## 3. Under-, Over-, and Autoimmunity and (Some) Autisms

There are several scientific reviews describing varied intersections between autism and immune functions [8]. Aside from looking at the important division that is the innate vs. adaptive immune system, specific manifestations of immune functions represent an important detail in determining the relationship(s) between autism and the immune system. Four potential scenarios come about: autism comorbid to (i) a primary immune deficiency (inborn), (ii) an acquired immune deficiency, (iii) an ‘overactive’ immune system (e.g., allergy or atopy), and (iv) autoimmunity (where the immune system attacks ‘self’). It is important to mention that such distinctions do not rule out more than one area of immune function being related to individuals or subgroups of cases of autism.

Preliminary evidence for a primary immune deficiency being present in some cases of autism has been reported. The presence of a specific polysaccharide antibody deficiency (SPAD) in a subset of children with autism provides initial evidence for such a relationship. SPAD reflects “*impaired antibody production against encapsulated organisms that are common causes of pneumonia, sinusitis, and ear infection*” [17]. Classified as rare and with the requirement for further replicative study, SPAD is seemingly over-represented alongside a diagnosis of some people with autism and could, for example, be linked to the previously discussed issue of viral titer chemistry related to rubella vaccination and autism. Other cases of common variable immune deficiency (CVID), characterised by low levels of antibodies and recurrent infections, have similarly been observed in some people with autism [18], albeit without population-level prevalence data at the time of writing and with such studies being based on only small participant numbers. There are multiple other routes to a primary immune deficiency being present that have not yet been suitably explored in the context of autism. Cases of inherited disorders causing immune deficiency, such as agammaglobulinemia (blocking the growth of mature B lymphocytes), have been observed [19], but so far, no population studies are available to estimate prevalence and ascertain whether such issues are potentially over-represented in autism more widely. Prior research attributed this to a shared genetic mechanism, proposing that the proximal portion of chromosome 4q contained genes responsible for both immunoglobulin production and cases of autism spectrum disorder. Various other inborn errors of immunity have been discussed in the general context of neurodevelopmental disorders [20]. Cumulatively, such inherited disorder links are important in the context that various inborn errors of metabolism (IEMs) are already a potentially important part of plural autisms [21].

DiGeorge syndrome, otherwise called 22q11.2 deletion syndrome, is a primary route for the presentation of syndromic autism (autism estimated in approximately 15–20% of cases) [22], where syndromic autism is defined as autism appearing alongside a known genetic condition. DiGeorge syndrome is also strongly linked to the presence of immunodeficiency disorders [23], thus implying that there may already be an innate tendency towards a relationship between some autisms and immune deficiency pathology in this context. This and a variety of other forms of syndromic autism offer further evidence for the likelihood of immune deficiency affecting the presentation of some of the autisms.

Acquired immune deficiency, characterised as immune deficiencies arising following either infection, illness, or use of certain pharmacotherapies, is perhaps a less well-developed area of autism research. Outside of immune complications arising from the human immunodeficiency virus (HIV), causing acquired immunodeficiency syndrome (AIDS), where little data currently exists specific to autism, several routes to acquired immune deficiency may be pertinent for some. Specific medicines that work to suppress immune functions, such as corticosteroids, have been used in the context of autism and specifically in the context of certain ‘types’ of autism (e.g., regressive autism) [24]. The observations that certain types of cancer may be more prevalent in relation to autism [25] also carry the associated risk arising from treatment options such as chemotherapy and their widely known effects on various immune functions. One of the more under-appreciated causes of immunodeficiency is particularly relevant to autism: malnutrition and the increasingly observed nutritional deficiencies that follow a restrictive diet for some people, particularly children, with autism [26]. Whilst the focus has been on the appearance of scurvy and multiple related nutritional diseases appearing alongside autism under such circumstances [27], it is notable that very restrictive diets will also potentially impact important elements like protein and vitamin intake, which will also affect immune functions. Likewise, one must also consider how natural ageing also affects immune functions and particularly the concept of immunosenescence [28], where older age means a greater susceptibility to infection, a less optimal immunisation response, and an increasingly pro-inflammatory state of immune functions. Again, whilst there is little longitudinal study of issues like immunosenescence in the context of autism, there are pockets of data highlighting how biological ageing may be ‘altered’ among some autism phenotypes [29], alongside findings suggesting that telomere and metabolic biochemistry [30] may potentially be affected and subsequently linked to immune system ageing.

Although ‘overactive’ is a poor description of the complexity of immune functions, it serves a purpose in denoting the effect that issues such as allergy and atopy seemingly have in relation to autism. Allergy means exposure to a particular substance, whether through ingestion, skin contact, or inhalation, and the subsequent release of immunoglobulin E (IgE) to that substance, typically involving some kind of cascading inflammatory response. One particularly notable cell type that seems to be intricately involved in allergy is the mast cell, which, through the process of degranulation, releases chemicals such as histamine and related inflammatory signalling compounds to the site of exposure. Studies specifically looking at IgE levels and autism have not, in the most part, shown any significant over-representation of issues outside of what would be typically expected [31]. When, however, atopic conditions such as skin and breathing conditions have been analysed, there do seem to be some important trends emerging for some [32]. So, in the context of over-representation of individual and family history of dermatitis and asthma [33], important connections are being made. Studies utilising Mendelian Randomisation techniques have even talked about ‘causal’ bidirectional effects between atopic conditions and autism [32]. Research looking at mast-cell-related issues, including mast cell activation disorder (MCAD), and autism has likewise highlighted several important connections [34] that require both greater study and offer relevant intervention options. We would also draw attention to the increasingly important body of work observing issues with non-IgE-mediated immune system issues that also seem to coincide with a diagnosis of autism. One important area in this realm is the finding of non-IgE-mediated food ‘intolerance’ that seems to affect some individuals diagnosed with autism [35]. This intersects with other data showing that specific exclusion diets may be a consideration for some on the autism spectrum [36] and, to some extent, overlaps with other research suggesting that a breakdown of various biological barriers coincident to autism may offer a route for the presentation of antigens to the immune system [37], particularly in the context of gastrointestinal (GI) immune processes.

Autoimmunity is a complicated manifestation of immune function where the immune system wrongly attacks healthy ‘self’ tissues or functions, resulting in local or systemic damage. Various inborn and acquired conditions are described as autoimmune, reflective of the intrinsic quality of a breakdown in central and peripheral immune tolerance to self. The intersection between autoimmunity and autism, although still preliminary, provides some of the strongest evidence of a relationship between immune functions and some cases of autism. Multiple lines of evidence discuss familial autoimmune conditions as potential risk factors for offspring autism [38]. Likewise, across the multitude of comorbid conditions known to be over-represented alongside autism, many feature an autoimmune element, covering conditions as diverse as type 1 diabetes, inflammatory bowel diseases, and coeliac disease [39]. The tenet ‘birds of a feather flock together’ also manifests in reports of multimorbidity of autoimmune conditions linked to autism presentation.

We cannot do justice to the huge bank of research suggesting overlaps between immune functions and some phenotypes of the plural autisms. We can, however, state that there is preliminary evidence for multiple roles for immune functions potentially intersecting with multiple different autisms, albeit with data tending to be based on small sample sizes, very selective clinical subgroups, and/or case study-based observations. Technological advances make future analyses of such immune system issues potentially standard, routine, and relatively inexpensive.

## 4. Models of Immune System Effects on (Some) Autisms: Maternal Immune Activation (MIA) and Maternal Autoantibody-Related (MAR) Autisms

We move to two specific areas where immune functions seemingly have an important role to play in both the aetiology and pathology of some cases of autism. Importantly, both models assume immune system effects acting on a developing child in utero and provide *prima facie* evidence for the notion of the plural autisms.

Maternal immune activation (MIA) typically refers to a state where maternal exposure to infection during pregnancy results in a cascade of biological processes with an immunological component to them, potentially affecting a developing child [40]. The starting point for the effects of MIA is that pregnancy is a time of revised immune tolerance to ensure that the maternal immune system does not perceive the developing foetus as foreign tissue. Examination of the effects of the seasonal influenza virus on offspring outcomes, such as the risk of schizophrenia, represents a beginning point for MIA. The combined results suggest the possibility of a connection, further confirmed by various animal model data [41]. Various gaps in the knowledge base remain about the effects of other confounding variables, both genetic and environmental, and the often-long latency period between MIA and the onset age of schizophrenia.

Stress is a potentially important driver of MIA in genetically susceptible mothers, which may also trigger an overexaggerated immune response. Within a developing foetus who is also vulnerable to this inflammatory state, the early concept of ‘a state of innate immunity priming’ emerges [42]. Prenatally and also postnatally, offspring develop an immune system primed to overreact to inflammatory stimuli such as pathogens and stress, producing an exaggerated innate immune response that has an impact on systemic inflammation and neuroinflammation. During critical windows of development, external immune triggers such as common bacterial and viral infections lead to exaggerated cytokine responses that, when triggered, are often difficult to switch off. Metabolic sequelae due to glycolytic switching from oxidative phosphorylation (the ‘Warburg effect’ [43]) are postulated to leave these children vulnerable to hypoglycaemia and mitochondrial stress. This stressed state has been examined by Lingampelly et al. [44], looking at metabolite profiles in newborns and 5 year olds in both autistic and non-autistic children. Said metabolic signatures potentially define a profile demonstrating loss of inflammatory mediators and a failure in the dampening of purinergic signalling, although one needs to exercise caution on this being correlative and not necessarily causative.

Clinically, this may be seen in young children with a profound developmental response to an immune trigger, whether presenting as neurodevelopmental regression or a period of static development. Whilst these children do not tend to come to early clinical attention, developmental regression is often described (language loss, for example), which, for many families, is not taken seriously by clinicians or is difficult to explain clinically in the absence of severe encephalopathy that would have, for example, required hospital admission.

With autism in mind, research has encountered similar obstacles when describing the effects of MIA, although perhaps benefiting from the (typically) earlier presentation of autism compared with schizophrenia. That various viral infections, such as rubella and cytomegalovirus (CMV) [45], experienced during pregnancy already also show a connection to the risk of autism in some offspring provides a template for the applicability of MIA to particular autism phenotypes, notwithstanding a role for other variables also potentially acting synergistically and causally. In terms of mechanisms of effect from MIA to the onset of a complicated condition like autism, science is still evolving. Cytokines, small proteins important in immune signalling, seem to have an important effect, whether in the upregulation of pro-inflammatory cytokines or a downregulation of more anti-inflammatory cytokines or some combination. Various other immune-related compounds have also provided food for thought. Timing of infection and immune system ‘exposure’ also seems to be an important variable, as does the biological sex of the developing offspring [46], illustrated by the still pronounced sex bias for conditions such as autism.

Maternal autoantibody-related (MAR) autism represents another autism phenotype. Distinct from the MIA model of autism, MAR autism involves a different branch of immune functions as being of central importance—autoimmunity, specifically autoimmunity towards foetal brain tissue [47]. Such antibodies, including collapsin response mediator proteins 1 and 2 (CRMP1 and CRMP2), which are detectable in maternal blood samples, cross the placenta and enter the foetal brain to alter brain development, contributing to autism. Screening for specific combinations of foetal brain antibodies has suggested that they are present in between 10 and 20% of maternal samples where offspring have autism [48]. Initial data on the expression of autism in children considered to have MAR autism also report elevated autism severity scores. Such research has now moved towards a commercial test for MAR autism alongside discussions on potential early intervention options.

Both the MIA autism model and MAR autism provide important templates for how immune functions can seemingly influence complex developmental and behavioural profiles. The data produced so far on these phenotypes, whilst important, is still in its infancy, not least regarding the specific mechanics affecting underlying biological processes and, again, with the requirement for larger-scale studies of prevalence across the autisms.

## 5. Expanding Immune Modulating Disease ‘Causing’ (Some) Autisms

Research linking infections such as rubella and CMV to autism risk and onset has been discussed. Both these conditions represent examples of how congenital infections—acquired by the mother and passed on to the developing child—can seemingly affect offspring behaviour and development. Such congenital infections, however, represent only a proportion of the various viral and bacterial pathways that can be acquired postnatally, which may potentially affect the risk of autism and associated neurodevelopmental conditions.

Vector-borne diseases such as malaria, transmitted by Anopheline mosquitoes, have shown correlations with rapid-onset autism in children presenting with typical developmental patterns prior to infection in a case series design study [49]. In such cases, autism typically follows a pattern of severe malaria, including high fever and seizures, which, in some cases, may also potentially fulfil criteria for cerebral malaria. This is followed by an often rapid regression of various facets of behaviour leading to a diagnosis of autism. There is little follow-up data about such cases, and whether such reports represent ‘lifelong autism’ or something less permanent in the context that a non-persistent pattern of autism presentation is something currently being researched [50]; questions about whether infection symptoms clearance might also be applicable to malarial autism are being raised too. The specific biological processes leading from malaria to autism have also not yet been fully elucidated. One would assume that in cases such as malaria involving the transmission of a parasite from host to prey, immunological processes, particularly inflammatory and pathogen clearance processes, would be an important part of the threat response.

In a similar vein of immune-provoking or immune-modulating diseases potentially driving autism aetiology for some, multiple reports have recorded instances of neuroinflammatory-linked conditions causing autism. Primary among such reports is a role for various types of encephalitis and meningitis. Enterovirus encephalitis—encephalitis caused by non-enveloped RNA viruses under the *Picornaviridae* family—has been linked to developing regression that leads to autism [51]. Enterovirus encephalitis is a rare complication following exposure to a common family of viruses linked to diseases as wide-ranging as the common cold to poliomyelitis. Individual reports and case series detailing the rapid onset of autism as an outcome following enterovirus encephalitis have been published. Including both ‘massive regression’ and, importantly, mentioning recovery following intervention, such cases highlight the potential role of acquired infections in some cases of autism onset and course, with some mimicking the infection–recovery cycle. Various other types of encephalitis have also been discussed in the context of the onset of some cases of autism, including herpes encephalitis (caused by the herpes simplex virus) [52] and observations of adult-onset autism being temporally correlated with infection. We have also previously discussed a role for autoimmune encephalitis (AE)-manifesting autism or autistic traits [53], and multiple reports of intervention options used in such cases also affect behavioural presentations. Precise details on how prevalent such infections and states are in autism are scarce, again reiterating the need for larger-scale studies despite their mention in the context of the ICD-11 description of autism [1].

Cumulatively, such research suggests an important link between immune-modulating disease and the presentation of some cases of autism [54]. The individual observations of a potential abatement of autistic and other behavioural traits following infection clearance and/or use of immune-modulating interventions for some strengthen such a link, albeit with cautions around inferred causality and notwithstanding other potential influences.

## 6. Immune-Altering Intervention Options and (Some) Autisms

Given the preliminary evidence describing a potential association between some of the plural autisms and immune functions alongside initial descriptions of the nature of immune system involvement, including infection, the question of personalised intervention arises. Research detailing the impact of rubella vaccination on reducing offspring autism risk in the context of congenital rubella syndrome has been discussed [12]. Given that immunisation aims to protect against particular pathogens and their deleterious effects, one could similarly argue that other elements of the vaccination schedule may already have had similar protective actions, particularly when disease complications potentially entail encephalitis or meningitis as outcomes and their specific links to the onset of some cases of autism. More direct immune-modulating intervention options have also been mentioned across the myriad findings related to autism. Such interventions tend to be more precisely targeted depending on immune effects following trends in relation to either excessive immune behaviour (autoimmunity and immune hyperactivity) or immunological deficiencies.

Whilst not yet presented as any formalised clinical recommendations, various intervention options have been described in the existing research literature. Prenatally, targeted therapy could be focused on maternal immune vulnerability, such as those with an already diagnosed autoimmune disease, where treatment can be better monitored and optimised. The risk of offspring with neurodevelopmental disorders is typically higher in mothers with poorly controlled autoimmune disease. Postnatally, families with a history of autoimmune disease and/or regressive autism could have closer neonatal follow-up and screening for metabolic differences (hypoglycaemia), particularly when unwell with bacterial or viral infections. At the onset of any developmental regression or static development, early medical review and investigation for an elevated inflammatory state and subsequent use of immune modulators could be initiated.

The use of intravenous immunoglobulin (IVIG) represents one of the most common immune-mediating agents for some autisms. Composed of pooled immunoglobulins or antibodies and part of the adaptive immune system response, IVIG already has multiple first-line uses across a broad range of immune-related responses and conditions. Dosage is an important part of IVIG therapy, where low doses tend to be used as replacement therapy under immunodeficiency conditions and higher doses provide both immunomodulatory and anti-inflammatory effects in cases of autoimmunity and related conditions [55]. The evidence for the use of IVIG in autism is cautiously promising under multiple scenarios [56], impacting positively on core autistic symptoms and being effective for underlying immune biochemistry, albeit with the need for further large-scale controlled studies. Importantly, various comorbid conditions overrepresented alongside a diagnosis of autism may also seemingly benefit from IVIG instigation with a similar need for further investigations. Observations on loss of clinical improvements following discontinuation of IVIG in relation to some cases of autism may also provide important clues on the underlying nature of immune dysfunction and the need for further detailed inspection of immune functions in the longer term. Further work is required, not least on specific mechanisms of IVIG effectiveness across different autisms and on potential side effects and adverse effects following IVIG usage. Whether subcutaneous immunoglobulin use [57] may also be a viable alternative to IVIG also remains to be investigated with specific regard to cases of autism.

Corticosteroid use as a way of suppressing immune functions is another intervention option already studied in the context of the plural autisms [24]. Again, following a pattern of clinical indication for various inflammatory and autoimmune conditions, steroid use in autism is finding a potential place [58] alongside some of the complicated immune biochemistry that some autism types seem to entail. Preliminary studies have indicated that corticosteroid use may show particular personalised usefulness in autism phenotypes that include regression as part of the clinical picture [59], something that could provide clues as to how immune functions follow, or potentially dictate, such developmental and behavioural presentation, albeit with the requirement for further controlled study.

The battery of immune-modulating medicines that can be lifesaving in the context of different types of encephalitis or related neuroinflammatory conditions has likewise received initial, limited autism research attention. Different treatment lines and schedules, when autoimmune encephalitis (AE) also manifests as autism or at least with autistic symptoms, provide potential clues to the underlying biology. IVIG and steroids are a major part of such treatment schedules and, where both AE and autistic traits remit, suggest further efficacy as potential intervention options for some. Use of second-line interventions for AE, such as rituximab, has received limited research attention in the specific context of autism [60], but again, a more detailed study is required.

Various other immune-related interventions have also been trialled in the context of autism [61]. This ranges from compounds such as the use of sulforaphane [62] and minocycline [63] to compounds such as suramin, impacting purinergic signalling [64]. The results, whilst promising, have not been universally positive, considering the potential need for more targeted use of such preparations in terms of potential best responders in the context of plural autisms and the need for more personalised medicine. Associated findings on various other compounds with secondary immune-modulating properties, such as vitamin D and N-acetylcysteine (NAC) (as an anti-inflammatory), with respect to the presentation of autism continue to garner interest. Other lifestyle changes may also have an immune-modulating role to play. Dietary changes based on specific immune-related conditions, such as coeliac disease being diagnosed alongside autism [65], are indicated. Alongside the slightly less well-defined area of non-coeliac gluten sensitivity (NCGS), a gluten-free diet may also be indicated [66] as it, and a milk-free diet, may be related to other non-IgE-mediated food allergies associated with autism [67].

Several immune-altering intervention options have been investigated in the context of autism, whether as specific interventions for autism or when autism appears alongside immune-related pathology. The evidence for their usefulness is, for the most part, promising but still requires further controlled study, particularly around best responders as a function of plural autisms and their potential multiple modes of action. All immune-altering interventions we have discussed currently reflect conceptual and research-informed approaches rather than formal clinical recommendations.

## 7. The Potential for Immune Screening in Autism?

**Figure 1:**
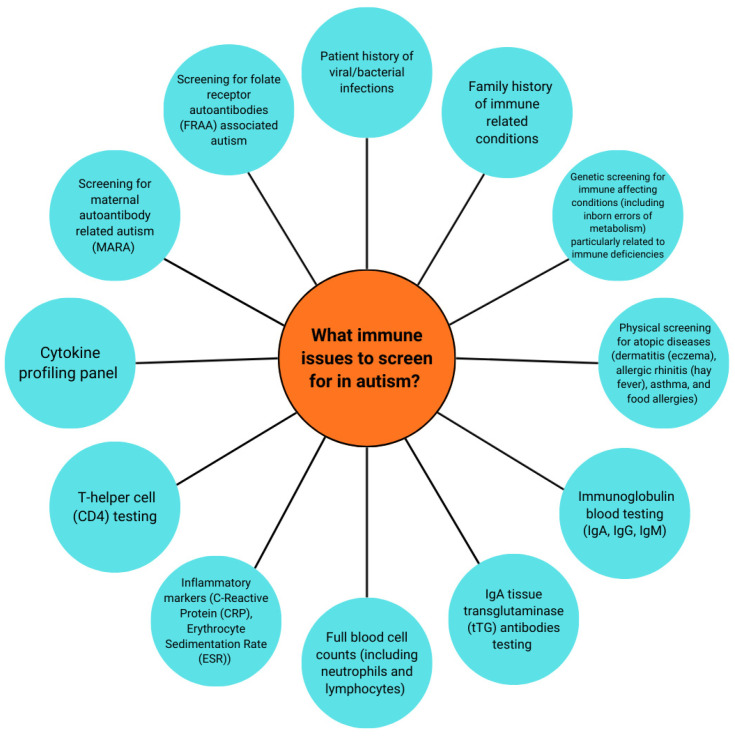
What potential immune issues could be screened for in autism?

Various intervention options have been suggested in the context of different immune issues presenting alongside some autism cases, including, for example, those affecting inflammatory processes [42]. Whilst not yet presented as any formalised clinical recommendations, the eventual choice of which intervention could be best suited to who will inevitably require informed guidance from appropriate screening and analysis of specific immune functions. The question therefore, in the context of any moves towards preferential screening for immune system issues when an autism diagnosis is being assessed for or has been made, is as follows: what immune system issues could be screened for? (see Figure 1).

Autism that is seemingly coincident with a viral or bacterial infection demands its own guidance when a serious, life-threatening illness is potentially involved. There are multiple guidelines already in place to diagnose and treat conditions such as encephalitis and meningitis and how important testing elements can and do inform treatment pathways. Children with recurrent infections who are exhibiting features of autism or ASD (even if not yet formally diagnosed) may potentially require screening for immune dysregulation or deficiency. A simple, inexpensive blood test evaluating immunoglobulin subtypes, for example, could potentially help rule out life-threatening primary immune deficiencies.

Where immune system issues alongside autism may be less apparent, there are several strategies that could eventually be utilised. Collecting a familial medical history is already an important part of autism assessment on the basis that various conditions, including autism, show high rates of heritability and/or familial transmission [68]. Such data is often also revealing in terms of the presence of immune-mediated conditions too, many of which bring high levels of heritability themselves and offer preferential early screening options. If, for example, a parent or sibling has received a diagnosis of coeliac disease, one would assume that preferential screening of other family members for such a gluten-related autoimmune condition would be merited based on heritability data [69]. Given also the evidence linking genetic predisposition between autism and immune-mediated pathologies, whole-genome sequencing (WGS) could likewise be a useful tool in this high-risk subgroup.

Similarly, physical examination of a child, for example, brought in for clinical assessment, can provide initial clues to potential underlying immune pathology. Atopic diseases manifesting as skin or respiratory conditions are relatively easy to screen for and may already be part of the clinical picture based on medication records. Such records can also reveal other signs of immune issues and conditions based on prescription patterns and more.

Routine blood tests typically offered in primary practice could represent a potential first line of screening. Full blood cell counts, examining red and white cells and platelets, may offer initial clues to the presence of infection or inflammatory processes, as well as picking up other over-represented issues in autism, such as anaemia [70], and potentially other nutrition markers such as vitamin D and zinc levels. Screening for specific inflammatory markers such as C-reactive protein (CRP) is also typically routine across many healthcare systems and can provide more specific information on inflammatory processes in the context of studies suggesting a link between CRP elevations and some autism presentations [71]. Other simple measures, such as the erythrocyte sedimentation rate (ESR), could likewise provide further data on inflammatory activity. This also represents an important first step in looking at the risk of inflammatory bowel diseases, such as Crohn’s disease, again in the context that such issues are potentially over-represented alongside a diagnosis of autism [72].

More targeted screening may also be indicated, some of which could be more routinely carried out based on the plurality of autism and the different biological routes that confer symptoms and a diagnosis. Although many countries already provide initial screening for inborn errors of metabolism (IEMs), the association between these and autism, and a wide variety of other genetic conditions, would suggest the possibility of extended screening based on their risk of conferring immune-related pathology, particularly immune deficiencies. As already mentioned, autism as a part of genetic syndromes such as DiGeorge syndrome may imply preferential analyses of immune functions. Analyses of other cases of syndromic autisms may also lead to similar screening based on what genetic condition is present and what links it may have to immune-related issues.

Direct screening for the presence of MIA and MAR autism, whilst advancing, is still in its infancy and typically requires analysis of maternal pregnancy biomarkers. Additional screening of immune functions could also be useful in such contexts. Earlier measurements of urinary or plasma metabolites showing potential differences in autistic children [44] may prove useful in the future following additional studies. Measures looking at neuroinflammation and neuroinflammatory processes, microglial activation, and cytokine chemistry, including T-helper cell chemistry and responses, may also prove useful for some, considering current findings with potential cases and subgroups of autism [73,74,75]. Other potential screening options not covered in our discussions, but nonetheless potentially research-rising and important to some, include specific analyses of folate receptor autoantibodies [76] previously linked to some cases of autism. Such results may also guide other intervention options, such as the use of folinic acid [77,78] and other dietary regimes (e.g., a milk-free diet considering molecular mimicry). All immune screening methods we discussed reflect conceptual and research-informed approaches rather than formal clinical recommendations.

## 8. Conclusions

Evidence gathered from our narrative review for both direct and secondary involvement of the immune system among specific types of the plural autisms is preliminary but gaining momentum. Small sample sizes, selective clinical subgroups, and/or case-based observations are currently reflective of most of the available research literature and represent important limitations to the current data and its extrapolation to autism. That many cases of autism are also accompanied by various over-represented comorbidities also complicates discussions on any autism-specific immune-related correlations. Existing evidence does, however, point to various immune system effects covering immune deficiency, atopy, exaggerated innate immunity, and autoimmunity as exerting potentially important influences on the aetiology and presentation of some cases of autism, either directly or through peripheral mechanisms such as comorbidity. Specific infections eliciting immune system activation, notably inflammatory effects, also show potential relationships with some autism phenotypes and, in some cases, may be potentially amenable to intervention via the use of personalised immune-related biologics and other treatments, albeit with the need for further study before any formal clinical recommendations can be made. The significant issues with the quality of the research evidence presented across multiple areas of immune system functioning in the context of autism require further large-scale research attention. Whilst we have presented ideas pertinent to an initial roadmap to potential further screening in this document, we stress that this remains purely conceptual, with a pressing need for the development of more standardised screening guidance for various immune system issues when a diagnosis of autism is sought and/or received and with appropriate personalised intervention offered. Likewise, there is a need for further research on how plural autisms manifest immune system issues and how such immune issues may themselves also further guide potential subgrouping of the heterogeneous autism spectrum.

## Data Availability

No new data were created or analyzed in this study. Data sharing is not applicable to this article.
